# A method to determine regional mechanical left ventricular dyssynchrony based on high temporal resolution short axis SSFP cine images

**DOI:** 10.1186/1532-429X-14-S1-W18

**Published:** 2012-02-01

**Authors:** Jonathan Suever, Brandon K Fornwalt, Michael Lloyd, John N Oshinski

**Affiliations:** 1 Wallace H. Coulter Department of Biomedical Engineering, Georgia Institute of Technology / Emory University, Atlanta, GA, USA; 2 Dept of Pediatrics and Biomedical Engineering, Division of Pediatric Cardiology, University of Kentucky, Lexington, KY, USA; 3 Dept of Cardiology, Division of Electrophysiology, Emory University, Atlanta, GA, USA; 4 Dept of Radiology & Imaging Sciences, Emory University, Atlanta, GA, USA

## Background

Left ventricular (LV) mechanical dyssynchrony has been proposed as a parameter to select patients for cardiac resynchronization therapy (CRT) [Bax et al JACC 2005].Several recent studies have shown that placing the LV pacing lead in the most delayed regions yields a better response to CRT [Ansalone et al JACC 2002]. However, most imaging-based methods assess global LV dyssynchrony providing a single value for the entire LV. *Regional* maps of LV dyssynchrony are required for planning LV lead placement.

The objective of this study was to develop a method to create a map of regional left ventricular mechanical dyssynchrony based on short-axis SSFP cine images.

## Methods

We examined a series of 13 patients that met standard criteria for CRT (QRS>120ms, NYHA HF class III-IV). Patients underwent a CMR exam prior to CRT that included acquisition of high temporal resolution short-axis cine images (60 frames/cardiac cycle). The endocardial boundary was delineated in a semi-automated manner, and the boundary contour was sampled at 100 equally spaced points (Figure 1A). A time-series of radial motion curves relative to the center of mass was generated for each radial location and over each slice.

## Results

To detect dyssynchronous regions of the LV, a model of “synchronous” contraction must be identified. Our method to identify a synchronous contraction curve used quality threshold (QT) clustering to identify the radial contraction curves that were most alike and most prevalent in the LV by measuring the “distance” between all of the curves to identify similar trajectories [Heyer et al Genome Research 1999]. The largest cluster of curves was considered to be representative of synchronous contraction of the left ventricle. All curves that belong to this representative cluster were averaged to obtain a reference curve.

Using cross-correlation, each radial contraction curve was shifted in time to find the *time shift* that resulted in the best correlation with the reference curve (Figure 1B). This analysis was repeated for every point throughout the LV and *time shift* values were projected onto the standard AHA 17-segment model to create a mechanical dyssynchrony map (Figure 1C). We were able to successfully generate dyssynchrony maps in all 13 patients.

## Conclusions

We present a robust method to provide a map of regional LV mechanical dyssynchrony that utilizes cross-correlation with a patient-specific reference contraction curve. This method can be used to identify regions of mechanical dyssynchrony for lead placement planning.

## Funding

This research was funded by the National Science Foundation Graduate Research Fellowship and the American Heart Association.

